# Pathogenic variants in *MT‐ATP6*: A United Kingdom–based mitochondrial disease cohort study

**DOI:** 10.1002/ana.25525

**Published:** 2019-07-01

**Authors:** Yi Shiau Ng, Mika H. Martikainen, Gráinne S. Gorman, Alasdair Blain, Enrico Bugiardini, Apphia Bunting, Andrew M. Schaefer, Charlotte L. Alston, Emma L. Blakely, Sunil Sharma, Imelda Hughes, Albert Lim, Christian de Goede, Meriel McEntagart, Stefan Spinty, Iain Horrocks, Mark Roberts, Cathy E. Woodward, Patrick F. Chinnery, Rita Horvath, Victoria Nesbitt, Carl Fratter, Joanna Poulton, Michael G. Hanna, Robert D. S. Pitceathly, Robert W. Taylor, Doug M. Turnbull, Robert McFarland

**Affiliations:** ^1^ Wellcome Centre for Mitochondrial Research Newcastle University Newcastle upon Tyne United Kingdom; ^2^ Faculty of Medicine University of Turku, and Division of Clinical Neurosciences, Turku University Hospital Turku Finland; ^3^ Medical Research Council Centre for Neuromuscular Diseases University College London Queen Square Institute of Neurology and National Hospital for Neurology and Neurosurgery London United Kingdom; ^4^ Department of Neuromuscular Diseases University College London Queen Square Institute of Neurology London United Kingdom; ^5^ Nuffield Department of Obstetrics and Gynaecology University of Oxford Oxford United Kingdom; ^6^ Royal Manchester Children's Hospital Central Manchester University Hospitals National Health Service Foundation Trust Manchester United Kingdom; ^7^ Department of Paediatric Neurology Royal Preston Hospital Preston United Kingdom; ^8^ South West Thames Regional Genetics Service St. George's Hospital London United Kingdom; ^9^ Alder Hey Children's National Health Service Foundation Trust Liverpool United Kingdom; ^10^ Greater Glasgow and Clyde National Health Service Yorkhill Hospital Glasgow United Kingdom; ^11^ Greater Manchester Neuroscience Centre Salford Royal National Health Service Foundation Trust, Manchester Academic Health Science Centre Salford United Kingdom; ^12^ Neurogenetics Unit National Hospital for Neurology and Neurosurgery London United Kingdom; ^13^ Department of Clinical Neurosciences University of Cambridge, Cambridge Biomedical Campus Cambridge United Kingdom; ^14^ MRC Mitochondrial Biology Unit University of Cambridge Cambridge United Kingdom; ^15^ Department of Paediatrics The Children's Hospital Oxford United Kingdom; ^16^ Oxford Medical Genetics Laboratories Oxford University Hospitals National Health Service Foundation Trust Oxford United Kingdom

## Abstract

Distinct clinical syndromes have been associated with pathogenic *MT‐ATP6* variants. In this cohort study, we identified 125 individuals (60 families) including 88 clinically affected individuals and 37 asymptomatic carriers. Thirty‐one individuals presented with Leigh syndrome and 7 with neuropathy ataxia retinitis pigmentosa. The remaining 50 patients presented with variable nonsyndromic features including ataxia, neuropathy, and learning disability. We confirmed maternal inheritance in 39 families and demonstrated that tissue segregation patterns and phenotypic threshold are variant dependent. Our findings suggest that *MT‐ATP6*–related mitochondrial DNA disease is best conceptualized as a mitochondrial disease spectrum disorder and should be routinely included in genetic ataxia and neuropathy gene panels. ANN NEUROL 2019;86:310–315

Mutations in *MT‐ATP6* are a recognized cause of maternally inherited mitochondrial DNA disease. Established syndromes of *MT‐ATP6*–related mitochondrial disease include Leigh syndrome (LS),^1^ and the syndrome of neuropathy, ataxia, and retinitis pigmentosa (NARP).^2^ Other presentations associated with *MT‐ATP6* mutations include a Charcot‐Marie‐Tooth (CMT) disease–like pure peripheral neuropathy^3^ and spinocerebellar ataxia (SCA) with upper motor neuron signs.^4^ However, the relative frequency of various presentations and features most suggestive of *MT‐ATP6* disease remains unclear. To elucidate the genotype–phenotype correlate of *MT‐ATP6*–related mitochondrial disease and associations with the underlying mutations, we sought to characterize *MT‐ATP6*–associated mitochondrial disease in a well‐characterized, large mitochondrial disease patient cohort.

## Patients and Methods

### *Subjects*

#### *Inclusion Criteria*

Subjects harboring pathogenic *MT‐ATP6* variants were identified from the National Health Service (NHS) Highly Specialised Service for Rare Mitochondrial Disorders (Newcastle, Oxford, and London, United Kingdom) and from the UK Mitochondrial Disease Patient Cohort (REC: 13/NE/0326) between January 2009 and June 2018. Diagnostic criteria used for LS was described elsewhere.^5^, ^6^ Carrier testing was offered to all maternal family members following genetic confirmation in the proband, and they were assigned as asymptomatic if the clinical assessment was normal. A standardized pro forma was used to capture clinical, radiological, neurophysiological, and molecular genetic data.

#### *Exclusion Criteria*

Previously unreported novel variants with unknown clinical significance were excluded from this study.

This study was approved and performed under the ethical guidelines and Declaration of Helsinki. Written informed consent for genetic testing was obtained from all participants.

### *Molecular Genetics and Measurement of Mutant Heteroplasmy*

*MT‐ATP6* and *MT‐ATP8* genes were screened by direct sequencing of polymerase chain reaction (PCR)‐amplified products as previously described.^7^, ^8^ Individual pathogenic *MT‐ATP6* variants were screened either by quantitative pyrosequencing or by fluorescent restriction fragment length polymorphism analysis, which permitted the quantitation of mtDNA heteroplasmy at the relevant nucleotide to a level of >3% heteroplasmy.^9^, ^10^

### *Statistical Analysis*

Descriptive statistical analysis was performed using Minitab (version 17.0; Minitab, State College, PA), SPSS (version 23.0; IBM, Armonk, NY), and R (version 3.5, R Foundation for Statistical Computing, Vienna, Austria). Nonparametric tests were performed to determine if there was any statistically significant difference between the different groups. The statistical significance was determined at ≤0.05. χ^2^ tests were performed to compare the proportion of variables between different categories, and the adjusted *p* value was reported where appropriate based on Bonferroni correction. A logistic progression model was used to evaluate the relationship of mutant blood heteroplasmy levels and individual risk of manifesting with disease, based on the methods previously described elsewhere.^11^, ^12^, ^13^

## RESULTS

### *Demographic Description*

We identified 125 individuals from 60 pedigrees harboring pathogenic *MT‐ATP6* variants. These included 88 clinically symptomatic individuals (39 female; median age at last follow‐up = 26.5 years, range = 0.75–74 years, interquartile range [IQR] = 33.3 years) and 37 asymptomatic family members (32 female; median age at last follow‐up = 40 years, range = 10–84 years, IQR = 23 years). Overall, the median age of disease onset was 3.75 years (range = 0–71 years, IQR = 16.9 years). Patients with LS had a significantly lower median age of onset compared to those without LS (1.5 vs 15 years, *p* < 0.001). Fifteen patients were deceased (median age = 20.5 years, range = 0.75–74 years, IQR = 26.6 years), and the survival status of 4 patients was unknown at the time of analysis.

### *Spectrum of Clinical Features*

Summative analysis of the available clinical data revealed that the most common clinical examination findings were cerebellar ataxia (60/72), followed by peripheral neuropathy (43/58) and learning disability (40/62). Mixed upper and lower motor neuron signs were identified in 34 individuals (34/63). Thirty‐one patients had a clinical phenotype compatible with LS (31/81), whereas just 7 patients manifested with the complete NARP phenotype. Among the patients who had muscle strength documented, distal neurogenic weakness was the most common pattern (13/53), closely followed by proximal neurogenic weakness (11/53). A mixed pattern of neurogenic muscle weakness was evident in 6 individuals (6/53), and the remaining patients had normal muscle power. Seizures were noted in 19 individuals (19/84), whereas dystonia was documented in 10 patients (10/81). The prevalence of clinical features and findings in patients harboring the 5 most common *MT‐ATP6* mutations are presented in the Table.

**Table 1:** Clinical Features and Findings Associated with the Five Most Common Pathogenic *MT‐ATP6* Variants

	m.8993T>C	m.8993T>G	m.9035T>C	m.9176T>C	m.9185T>C
Demographic data
No. of patients	24	22	8	11	18
F/M	10/14	8/14	5/3	6/5	7/11
No. of pedigrees	20	19	3	5	9
No. of deceased	4	5	1	2	3
Median age, yr (range, IQR)	27.5 (3–74, 38.8)	30 (0.75–59, 39)	24 (10–48, 23)	15.5 (2–49, 19.5)	25 (19–54, 29)
Median age of onset, yr (range, IQR)	5.5 (0.5–71, 22.3)	2 (0–34, 11.1)	10 (3–19, 15.3)	1 (1–32, 3.9)	6 (2–15, 8)
Clinical findings
LS	8/23	11/17	2/8	6/11	3/18
UMN signs	9/20	10/14	4/8	6/10	10/16
Learning disability	14/18	6/8	5/7	5/9	9/16
Seizures	6/22	9/20	0/8	3/8	0/18
Dystonia	3/24	3/20	1/8	3/10	0/17
Ataxia	20/22	10/11	8/8	6/10	12/17
Neuropathy^a^	15/17	4/6	3/7	5/10	14/14
Pes cavus	9/22	1/12	2/3	4/11	7/12
RP^b^	3/18	12/13	2/7	1/9	0/13
Cardiac	2/17	3/9	0/4	2/8	0/11
DM	0/22	1/14	0/6	1/11	1/11
MRI head changes
Cerebellar atrophy	9/14	7/13	4/7	1/8	5/10
BG changes	8/14	8/13	1/7	3/8	3/10
Brainstem	5/14	0/13	1/7	2/8	0/7

Denominator values vary due to missing data.

a Reports of the nerve conduction studies were available for 26 patients. The most common finding was axonal, sensory‐motor neuropathy (23/26), followed by mixed axonal and demyelinating neuropathy (2/26), and only a single patient with the m.8993T>C variant had demyelinating neuropathy.

b χ^2^ test (Bonferroni correction; *p* ≤ 0.006) showed a higher proportion of patients with the m.8993T>G mutation had RP compared to patients harboring either the m.8993T>C (92% vs 17%, *p* < 0.001) or m.9176T>C (92% vs 11%, *p* = 0.001) variants.

BG = basal ganglia; DM = diabetes mellitus; F = female; IQR = interquartile range; LS = Leigh syndrome; M = male; MRI = magnetic resonance imaging; RP = retinitis pigmentosa; UMN = upper motor neuron sign defined as the presence of pathological brisk reflexes and/or positive Babinski sign.

Acute metabolic and physical decompensation during intercurrent illness was documented in 27 patients (27/60). Four adult patients (m.8993T>C, n = 3; m.9185T>C, n = 1) experienced episodic, abrupt disease exacerbations in the form of a sudden Leigh‐like crisis with worsening ataxia, and brainstem signs and symptoms including ophthalmoplegia, dysphagia, and cardiorespiratory disturbance, with corresponding subacute magnetic resonance imaging (MRI) signal abnormalities in the brainstem, thalamus, and cerebellum.

The profile of clinical features was compared between patients with and without LS (adjusted *p* value ≤0.003). Episodic metabolic decompensation (21/23 vs 6/36, *p* < 0.001), learning disability (18/19 vs 21/39, *p* = 0.002), and basal ganglia lesions (19/23 vs 5/30, *p* < 0.001) were significantly more common in patients with LS compared to those without LS. However, other clinical features such as neuropathy (11/15 vs 31/42, *p* = 1), ataxia (18/21 vs 36/45, *p* = 0.738), retinitis pigmentosa (RP) (5/15 vs 11/43, *p* = 0.738), seizures (10/31 vs 6/49, *p* = 0.044), and bulbar symptoms (11/13 vs 16/28, *p* = 0.156) were similarly present in both groups.

### *Neuroimaging Changes*

MRI head data were available for analysis in 53 clinically affected individuals. Symmetrical basal ganglia lesions and brainstem signal abnormalities were identified in 23 patients and 8 patients, respectively; 5 patients who did not fulfil the diagnostic criteria of LS had signal changes in the basal ganglia. Global cerebellar atrophy was identified in 24 patients. Strokelike lesions involving the occipital lobe and cerebellar cortex were identified in 1 patient.

### *Molecular Genetics*

We identified 9 previously reported pathogenic variants in our cohort of patients. The most common point mutation was m.8993T>C (27%), followed by m.8993T>G (25%), m.9185T>C (20%), m.9176T>C (13%), and m.9035T>C (9%). We were able to establish maternal transmission in 68 patients (77%, 39 families) and that the mutation likely arose de novo in 3 patients (3%). Maternal DNA samples were not available in 17 patients (20%).

**Figure 1:**
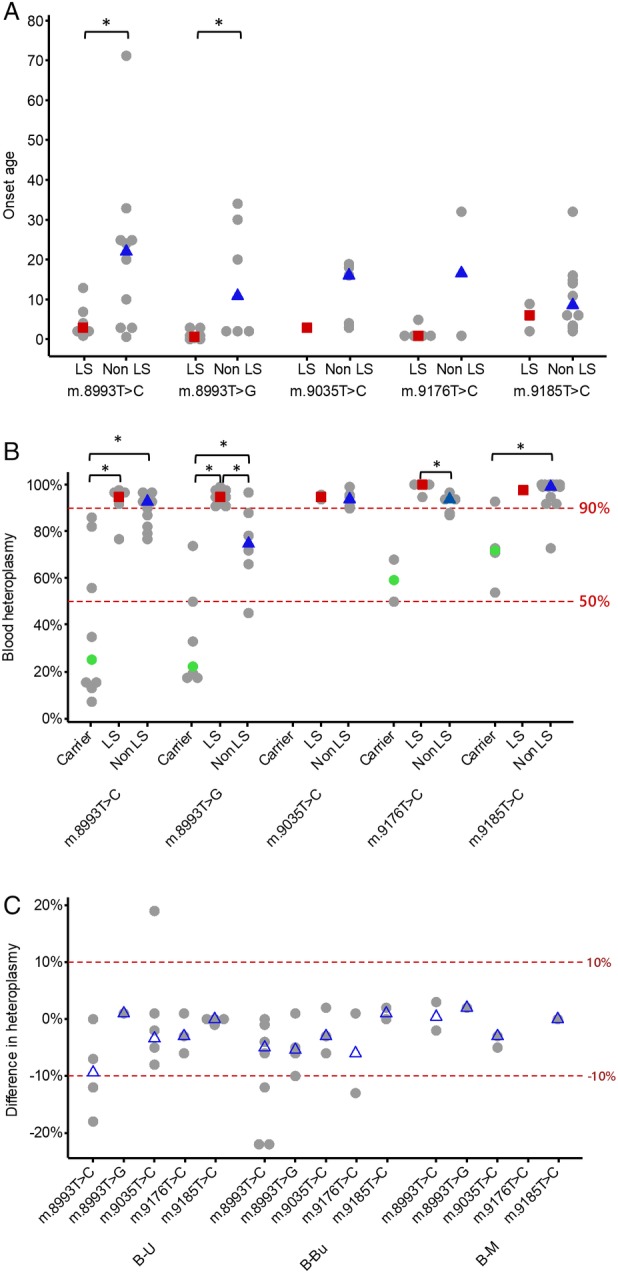
Molecular genetic data. (A) Individual dot plot showing the age of disease onset in patients harboring 5 common *MT‐ATP6* pathogenic variants. Grey circles represent individual patient data, the red squares represent the median blood heteroplasmy level for LS, and the blue triangles indicate the median blood heteroplasmy level for non‐LS. *p < 0.05 (Wilcoxon test). (B) Individual dot plot showing the variations in blood mutant heteroplasmy levels in 3 phenotypic categories (asymptomatic carriers, LS, and non‐LS) and *MT‐ATP6* pathogenic variants. Grey circles represent individual patient data, green circles represent the median blood heteroplasmy level in asymptomatic carriers, the red squares represent the median blood heteroplasmy level for LS, and the blue triangles indicate the median blood heteroplasmy level for non‐LS. *p < 0.05 (Wilcoxon test). We have examined the correlation of mutant heteroplasmy level and age of disease onset for each of the common *MT‐ATP6* pathogenic variants. There is no statistical significant correlation identified in any variants. (C) Individual dot plot showing the difference in mutant heteroplasmy levels across different *MT‐ATP6* variants. Grey circles represent individual patient data, and the blue triangles indicate the median difference of heteroplasmy level. B‐Bu = difference in the heteroplasmy level between blood and buccal samples; B‐M = difference in the heteroplasmy level between blood and muscle samples; B‐U = difference in the heteroplasmy level between blood and urine samples; LS = Leigh syndrome.

The age of onset and pathogenic mtDNA heteroplasmy levels in blood were compared across different pathogenic variants, as shown in Figure 1A and B. The variability in the mutant heteroplasmy level between different tissues (blood, urinary epithelial cells, and buccal mucosal cells) was typically <10% in *MT‐ATP6* variants (Fig 1C) except in m.8839G>C (26%, 76%, and 58% in blood, urine, and buccal samples, respectively), m.9032T>C (25%, 59%, and 96% in blood, urine, and muscle, respectively), and m.9134A>G (43% and 90% in blood and urine, respectively).

### *Risk of Disease Manifestation and mtDNA Heteroplasmy Level*

**Figure 2:**
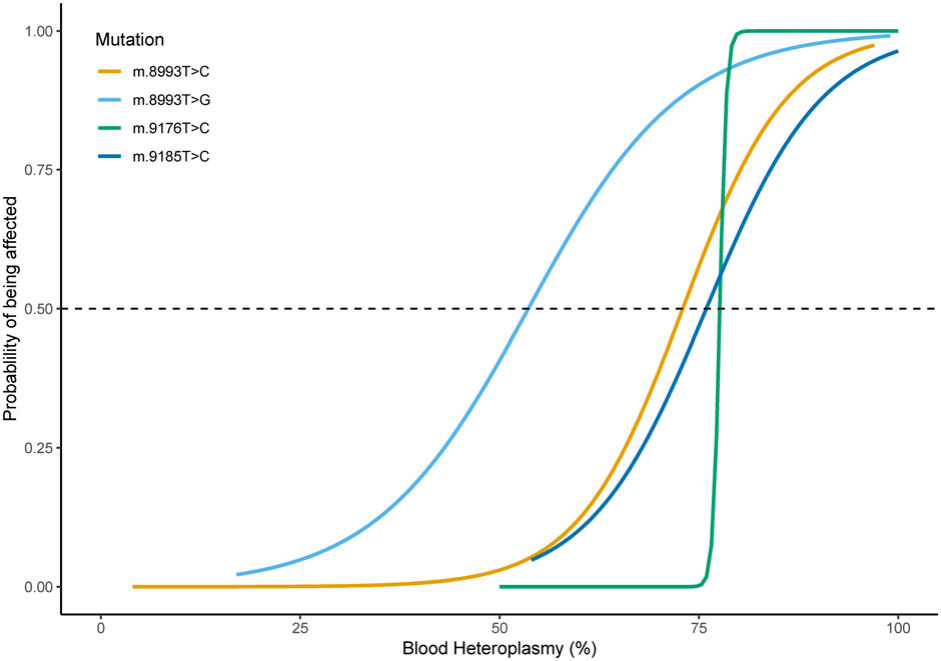
Risk of disease manifestation and blood heteroplasmy level. Estimated probability of being clinically affected based on the blood heteroplasmy level for 4 *MT‐ATP6* pathogenic variants (m.8993T>C, m.8993T>G, m.9176T>C, and m.9185T>C) is illustrated. For instance, for an estimated probability of 0.5 being clinically affected, the mutant heteroplasmy appears to be the lowest in the m.8993T>G variant (54%), compared to 3 other variants m.8993T>C, m.9176T>C, and m.9185T>C (73%–78%).

Our logistic regression analysis for the 4 common pathogenic variants was performed and showed that the m.8993T>G was associated with the lowest clinical expression threshold followed by the m.8993T>C, m.9185T>C, and m.9176T>C variants (Fig 2). The 95% confidence interval was not constructed individually for these variants due to the limited number of patients.

## Discussion

A recent review of 218 previously reported cases of 19 pathogenic *MT‐ATP6* variants highlighted the marked variations in the biochemical defect and phenotypic heterogeneity.^14^ This study showed the correlation between pooled pathogenic heteroplasmy and disease onset and severity. However, only 1 of the 14 new cases reported by Ganetzky et al had a confirmed pathogenic variant according to the American College of Medical Genetics criteria, illustrating the difficulties posed in confirming novel genetic diagnoses of mtDNA disease.^14^ Although some of our findings are aligned to those of Ganetzky et al, there are important additional aspects to our study that allow us to be more authoritative in our conclusions. These include the study design (a national cohort study with standardized clinical evaluation in 3 major referral centers), a detailed description of the neurological features (rather than a simple syndromic classification), and the novel findings of tissue segregation patterns and variant‐dependent phenotypic threshold.

In this national cohort study, we observed a continuum of clinical features in the *MT‐ATP6–*related mitochondrial disease. Cerebellar ataxia and axonal neuropathy were the most common features among these patients, often associated with some degree of learning disability. We identified that patients with LS may also exhibit overlapping features of NARP. Despite these common characteristics, we observed some emerging patterns associated with specific *MT‐ATP6* mutations. RP was most prevalent in m.8993T>G‐related mitochondrial disease compared to other *MT‐ATP6* variants; however, RP was only clinically identified in less than a third of all patients. All patients with the m.9185T>C mutation manifested with predominantly axonal, sensory‐motor neuropathy, but none had RP, dystonia, or seizures.

The peculiarities of mitochondrial genetics, namely heteroplasmy and threshold effect, are eloquently demonstrated in this cohort of patients.^15^ Our results demonstrate that phenotypic expression of the m.8993T>G mutation appears to have the lowest threshold level compared to other *MT‐ATP6* variants. Leigh syndrome appears to manifest at a high threshold level (≥90%), whereas other clinical phenotypes are associated with a lower mutant heteroplasmy, consistent with a previous observation.^16^ Several other *MT‐ATP6* pathogenic variants, including m.9035T>C, m.9176T>C, and m.9185T>C, are associated with a very high phenotypic threshold level (>90%). One of the most interesting findings is that once such threshold levels are breached, it is not possible to predict the clinical phenotype and disease severity based solely on the mutant heteroplasmy level. Moreover, the mutant loads of m.8993T>C, m.8993T>G, and m.9185T>C overlap in some patients with nonsyndromic neurological manifestation and in asymptomatic individuals. These findings have important implications not only for presymptomatic carrier testing but also discussion around reproductive options.^17^

Interestingly, of the 27 patients who experienced episodes of (sub‐)acute deterioration of their functional status during a febrile illness, 4 adult patients did not have a preexisting diagnosis of LS yet experienced severe brainstem disturbance. These serious neurological sequelae emphasize the need for early recognition of potential life‐threatening complications and instigation of timely supportive care, irrespective of the prevailing initial clinical phenotype. Our results also demonstrate that strokelike episodes are rare in *MT‐ATP6* mutations, corroborated with the observation of a smaller case series.^16^

There are several diagnostic caveats associated with *MT‐ATP6–*related mitochondrial disease compared to other common mtDNA mutations. Chronic progressive external ophthalmoplegia and systemic involvements, such as diabetes mellitus and cardiac abnormalities, are uncommon in *MT‐ATP6* variants compared to other mtDNA mutations.^18^, ^19^ Moreover, histochemical analysis of muscle biopsy and conventional respiratory chain analysis (complex I‐IV) are usually unremarkable in patients with pathogenic *MT‐ATP6* mutations, imposing the diagnostic challenge of validating the pathogenicity of rare or novel variants in clinical practice.^14^ On the other hand, the clinical presentation of common pathogenic *MT‐ATP6* variants may overlap with other hereditary conditions such as CMT or SCA.^4^, ^5^

In conclusion, we suggest that *MT‐ATP6*–related mtDNA disease is best defined as a mitochondrial disease spectrum disorder that includes core clinical features of cerebellar ataxia, peripheral neuropathy, and learning disability, with or without a Leigh‐like phenotype. Our findings highlight the importance of including *MT‐ATP6* gene sequencing in the gene panels of spinocerebellar ataxia and hereditary neuropathy. Moreover, the patterns of tissue segregation and variability in the phenotypic threshold have important implications for the genetic counseling and risk prediction of disease development.

## Author Contributions

Study concept and design: Y.S.N., M.H.M., G.S.G., D.M.T., and R.M. Data acquisition and analysis: all authors. Drafting the manuscript and figures: Y.S.N., M.H.M., G.S.G., A.Bl., D.M.T, R.M.

## Potential Conflicts of Interest

Nothing to report.
